# Deciphering the role of short-chain fatty acids (SCFAs) in diabetic nephropathy: protective or pathogenic mediators?

**DOI:** 10.1097/MS9.0000000000003883

**Published:** 2025-09-12

**Authors:** Kalsoom Yaseen, Muddassir Khalid, Aqsa Asif, Mohammed Hammad Jaber Amin

**Affiliations:** a Department of Medicine, Nishtar Medical University, Multan, Pakistan; b Department of Medicine, University Medical and Dental College, Faisalabad, Pakistan; c Department of Medicine, Alzaeim Alazhari University, Khartoum, Sudan

*Dear Editor,*

Diabetic nephropathy is a chronic inflammatory disease that develops as a complication of prolonged diabetes mellitus and is the primary cause of end-stage renal disease. Oxidative stress and NF-κB signaling activation play crucial roles in the pathogenesis of diabetic nephropathy^[1]^. Short-chain fatty acids (SCFAs), primarily acetate, propionate, and butyrate, produced from dietary fiber fermentation in the GIT, exert positive regulatory effects on inflammation and kidney injury^[2]^. SCFAs reduce inflammation, regulate blood pressure, increase insulin sensitivity, and decrease oxidative stress and NF-κB signaling through the activation of GPR41 and 43, thus acting as protective agents in diabetic nephropathy. These outcomes are consistent with a study showing SCFA-treated diabetic mice were protected from nephropathy^[3]^. Besides this, SCFAs, mainly butyrate, being a natural histone deacetylase inhibitor, make the gut barrier better, fuel the colonocytes, and reduce uremic toxins and albuminuria. Butyrate promotes the acetylation of histone, which is crucial for activating anti-inflammatory and anti-fibrotic agents that protect renal tubular cells and podocytes under hyperglycemic conditions. In clinical investigations and animal models, the increased intake of dietary fibers or SCFA administration has also been recognized to possess a protective effect in type 1 diabetes and type 2 diabetes due to their inhibitory effects on proinflammatory cytokines and reactive oxygen species^[4]^.

Another study shows that exogenous SCFAs, especially butyrate, ameliorate hyperglycemia, and insulin resistance, improve renal function, and restore hyperglycemia-induced inflammatory damage. These outcomes are the consequence of GPR43-β-arrestin-2 signaling, hence contributing to buffering oxidative stress and blocking NF-κB activation^[5]^.

However, the role of acetate is ambiguous in diabetic nephropathy, as propionate and butyrate are harmful in high doses as well. In diabetes, acetyl-CoA synthetase 2 converts acetate into acetyl-CoA, causing overexpression of fibrotic and inflammatory agents. They cause podocyte and renal tubular cell injury, which worsens the diabetic nephropathy. On the opposite side, a study demonstrates that acetate ameliorates diabetes-induced nephrotoxicity, which is associated with suppressed XO and its accompanying pro-inflammatory mediators^[6]^. Nevertheless, exogenous SCFAs and probiotics have been shown by studies to increase insulin sensitivity, fat decomposition, and body metabolism. Probiotic-based interventions increase the levels of SCFAs in the body that promote insulin sensitivity and fat decomposition, reduce inflammation, protect renal tubular cells, and prevent podocyte injury. To conclude, SCFAs, such as acetate, would be a promising diet-derived therapeutic agent, but research and randomized controlled trials are needed to elucidate the mechanisms and validate the protective or pathogenic nature of SCFAs.

TITAN Guidelines: This manuscript complies with TITAN Guidelines, 2025, declaring no use of artificial intelligence^[7]^.

## Data Availability

Not applicable.
